# Determinants of early neonatal mortality in Afghanistan: an analysis of the Demographic and Health Survey 2015

**DOI:** 10.1186/s12992-018-0363-8

**Published:** 2018-05-09

**Authors:** Gulam Muhammed Al Kibria, Vanessa Burrowes, Allysha Choudhury, Atia Sharmeen, Swagata Ghosh, Arif Mahmud, Angela KC

**Affiliations:** 1 Department of International Health, Johns Hopkins Bloomberg School of Public Health, Baltimore, MD-21205 USA; 2 Morgan State University, Baltimore, MD-21251 USA; 3 Department of Epidemiology, Johns Hopkins Bloomberg School of Public Health, Baltimore, MD-21205 USA; 4 University of Saskatchewan, Saskatoon, Saskatchewan Canada

**Keywords:** Demographic and health survey, Early neonatal mortality, Determinants, Afghanistan

## Abstract

**Background:**

Neonatal mortality is declining slowly compared to under-five mortality in many developing countries including Afghanistan. About three-fourths of these deaths occur in the early neonatal period (i.e., the first week of life). Although a number of studies investigated determinants of early neonatal mortality in other countries, there is a lack of evidence regarding this in Afghanistan. This study investigated determinants of early neonatal mortality in Afghanistan.

**Methods:**

Data from the Afghanistan Demographic and Health Survey 2015 (AfDHS 2015) were analyzed. After reporting the weighted frequency distributions of selected factors, a multilevel logistic regression model revealed adjusted associations of factors with early neonatal mortality.

**Results:**

A total of 19,801 weighted live-births were included in our analysis; 266 (1.4%) of the newborns died in this period. Multivariable analysis found that multiple gestations (adjusted odds ratio (AOR): 9.3; 95% confidence interval (CI): 5.7–15.0), larger (AOR: 2.9; 95% CI: 2.2–3.8) and smaller (AOR: 1.8; 95% CI: 1.2–2.6) than average birth size, maternal age ≤ 18 years (AOR: 1.8; 95% CI: 1.1–3.2) and ≥ 35 years (AOR: 1.7; 95% CI: 1.3–2.3), and birth interval of < 2 years (AOR: 2.6; 95% CI: 1.4–4.9) had higher odds of early neonatal mortality. On the other hand, antenatal care by a skilled provider (AOR: 0.7; 95% CI: 0.5–0.9), facility delivery (AOR: 0.7; 955 CI: 0.5–0.9), paternal higher education level (AOR: 0.7; 95% CI: 0.5–1.0), living in north-western (AOR: 0.3; 95% CI: 0.1–0.6), central-western regions (AOR: 0.5; 95% CI: 0.3–0.9) and in a community with higher maternal education level (AOR: 0.4; 95% CI: 0.2–0.9) had negative association.

**Conclusions:**

Several individual, maternal and community level factors influence early neonatal deaths in Afghanistan; significance of the elements of multiple levels indicates that neonatal survival programs should follow a multifaceted approach to incorporate these associated factors. Programs should focus on birth interval prolongation with the promotion of family planning services, utilization of antenatal care and institutional delivery services along with management of preterm and sick infants to prevent this large number of deaths in this period.

## Background

The past decades have seen rapid developments in reducing under-five mortality; however, neonatal mortality reduction remains a major challenge for most developing countries. Of the estimated 5.9 million under-five children who died globally in 2015, neonates comprised about 45% of the deaths [1, 2]. Between 1990 and 2015, under-five mortality declined by 53%, but the reduction of neonatal mortality in the same period remains slow as neonatal mortality declined by 47% compared to the 58% reduction of post-neonatal under-five mortality [2]. Reduction of neonatal mortality would be important to meet both the neonatal and under-five mortality targets of the sustainable development goals (SDGs). The SDGs have two specific targets to reduce the neonatal mortality rate (NMR) and under-five mortality rate (U5MR) to 12 and 25 per thousand live-births within 2030, respectively [3]. Achievement of these targets is not possible without reduction of neonatal mortality in countries with higher rates, specifically in developing countries where 98% of the neonatal deaths occur [2]. Most of the developing countries have a higher NMR including Afghanistan [1, 2] with an estimated NMR of 22 per 1000 live-births in 2015 [4].

Afghanistan is a landlocked country in South Asia with a land mass of about 652,230 km^2^ and an estimated total population of 34 million [5]. Similar to other developing countries, neonatal mortality declined slowly in this region compared to under-five mortality, with an annual rate of reduction (ARR) of 1.5% from 53 to 36 per thousand live-births in 1990 and 2015, respectively [2]. An acceleration of this slow ARR is required to achieve the neonatal mortality targets of the SDGs.

Recent global estimates suggest that about three-fourths of the neonatal deaths could be prevented by an increased coverage of currently available interventions [6, 7]. It is also well-established from a number of studies that about three-fourths of neonatal deaths occur during the early neonatal period (i.e., the first week of life) [8, 9]; this high proportion of deaths in this period indicates that the first week of life is the most critical period for neonatal survival. Similar to other developing countries, about three-fourths of neonates die in the early neonatal period in Afghanistan; this proportion is about one-third of the total under-five deaths [4]. Reduction of these preventable deaths in this period is crucial to meet the targets of SDGs [2].

Earlier research from other countries also demonstrated that early neonatal mortality is influenced by several maternal (e.g., age and parity), neonatal (e.g., birth weight and gender), and household and socioeconomic risk factors (e.g., parental education and wealth status) [10–13]. However, there remains a lack of evidence on determinants of early neonatal mortality in Afghanistan. This limits our understanding of this problem for an evidence-based programming and indicates that this issue has been underestimated in this country. We made an attempt to identify and fill this existing knowledge gap in Afghanistan. In this manuscript, we examined the determinants for early neonatal mortality in this country by using a nationally representative dataset. Our results could be useful in assisting policymakers and researchers to develop efficient strategies to improve survival of newborns in Afghanistan.

## Methods

### Data source

We used data from the Afghanistan Demographic and Health Survey (AfDHS 2015) to investigate determinants of early neonatal mortality. The AfDHS 2015 was the first DHS implemented in Afghanistan. It was a part of the global DHS Program. The survey was conducted from June 2015 to February 2016, and primarily provided estimates of basic demographic and health indicators of the country. It used an updated version of the ‘Household Listing Frame’ as the sampling frame. Details of this population-based survey including survey design, methodologies, sample size calculation and questionnaires have been described elsewhere [4].

### Sample design

A stratified two-stage sample design was followed in AfDHS 2015 to allow estimates of key indicators at the national level, in urban and rural areas, and for each of the 34 provinces of the country. During the first stage, the sample points (i.e., clusters) consisting of EAs were selected. A total of 950 clusters were selected in this stage; 260 and 690 separately for urban and rural areas, respectively. Some of the regions (10%) were difficult to reach due to security reasons; 101 reserve clusters were selected in all of the provinces to replace the inaccessible clusters [4].

### Data collection

The AfDHS used three questionnaires: women’s, men’s, and household’s questionnaires. All ever-married women with 15–49 years of age living in the households were eligible to be interviewed. They were interviewed using the women’s questionnaire. During the interview, several types of information were collected. These included: background characteristics (including age, education, and media exposure), birth history and child mortality, knowledge and use of family planning methods, fertility preferences, antenatal, delivery, and postnatal care, breastfeeding and infant feeding practices, vaccinations and childhood illnesses, marriage, women’s work and husbands’ background characteristics, awareness and behavior regarding HIV/AIDS and other sexually transmitted infections (STIs), adult and maternal mortality, knowledge, attitudes, and behavior related to other health issues (e.g., tuberculosis, hepatitis, fistula) and domestic violence (questions asked of one woman per household) [4].

### Coverage of the sample

In total, 25,741 households were selected for the AfDHS, of which 24,941 were occupied during the survey fieldwork. Of the occupied households, 24,395 were successfully interviewed with 98% response rate. In the interviewed households, 30,434 ever-married women age 15–49 were identified for individual interviews; interviews were completed with 29,461 of these women, yielding a response rate of 97% [4]. Overall, the survey was successfully carried out in 956 clusters [4].

### Participants

Among the 29,461 interviewed women, we examined the determinants of early neonatal mortality among the live-births of a cohort of women who had at least one live-birth within four years preceding the survey. A total of 19,636 women delivered at least one child within this period. We included their most recent live-births.

### Conceptual framework

**Fig. 1:**
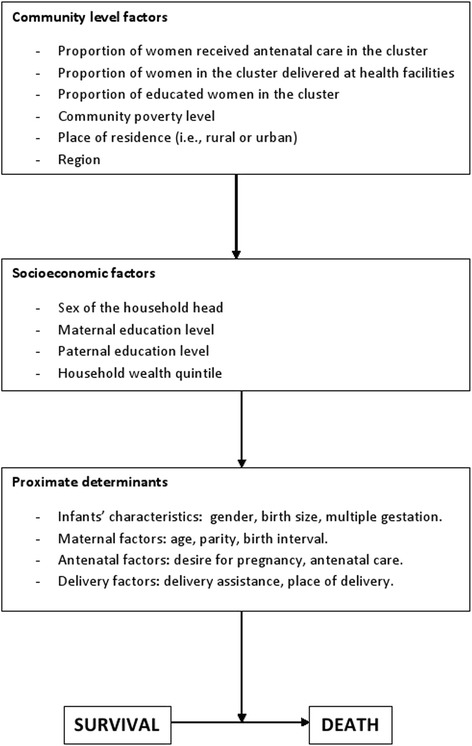
Conceptual Framework for determinants of early neonatal mortality. Adapted from Mosley and Chen’s framework for child survival in developing countries [14]

Figure 1 shows the modified conceptual framework that was adapted from Mosley and Chen’s framework for survival of children in developing countries [14]. This framework was modified based on published reports, limitations and the structure of the AfDHS dataset. Variables of the first two levels were considered individual level, which were nested within the community (i.e., cluster) where an infant lived.

### Study variables

The outcome variable for this study was early neonatal death, defined by a binary variable as the death of a live-born baby within the first week of life. The survived mothers were interviewed about the day of deaths of their children. The aggregated individual level characteristics of the community level were used to construct community level variables. The high and low categorization of the aggregate variables was done based on the distribution of the proportion values calculated for each community. Table 1 shows the list of the included explanatory variables (i.e., risk factors for neonatal deaths) along with their definitions and categories.

**Table 1:** Explanatory variables

Study variables	Description and categories
Infants’ individual factors	
Sex	Sex of the baby at birth (0 = female; 1 = male).
Birth size	Subjective assessment of the mother about child-size after birth (1 = average; 2 = smaller than average; 3 = larger than average).
Multiple gestation	Whether more than one child was born (0 = no; 1 = yes).
Maternal factors	
Maternal age	Maternal age during child-birth (0 = 19–34 years; 1 = ≤18 years; 2 = 35–49 years).
Birth rank	Birth order of the baby (0 = second to fourth pregnancy; 1 = primi; 2 = fifth or higher pregnancy).
Birth interval	Interval between last pregnancy and current pregnancy (0 = ≥2 years; 1 = < 2 years).
Antenatal factors	
Desire for pregnancy	Intention to become pregnant when women conceived (1 = wanted then; 2 = wanted later; 3 = wanted no more).
Antenatal care	Whether the mother received antenatal checkup during pregnancy by a skilled health care personnel (0 = no; 1 = yes).
Delivery factors	
Delivery assistance	Assistance during child-birth (0 = unskilled birth attendant; 1 = skilled delivery attendant). Skilled attendants were doctors, nurses, midwives, or auxiliary nurse/midwives.
Place of delivery	Place of child-birth (0 = home; 1 = hospital/health centre).
Socioeconomic characteristics	
Sex of the household head	Sex of the head of the household (1 = male; 2 = female).
Maternal education level	Education level of the mother (0 = no formal education; 1 = primary; 2 = secondary or higher).
Paternal education level	Education level of the father (0 = no formal education; 1 = primary; 2 = secondary or higher).
Wealth status	Household wealth quintile (1 = poorest; 2 = poorer; 3 = middle; 4 = richer; 5 = richest).
Community level factors	
Community antenatal care utilization	Proportion of women who received four or more antenatal care visit during pregnancy (1 = low (≤25%), 2 = high (> 25%)).
Utilization of facility delivery	Percentage of women in a cluster who were delivered at health facility (1 = low (≤50%), 2 = high (> 50%)).
Community education level	Percentage of women in a cluster who had at least secondary or above level of education (1 = low (≤50%), 2 = high (> 50%)).
Community poverty level	Percentage of women who were from the first two wealth quintiles (1 = high (≥75%), 2 = low (< 75%)).
Place of residence	Type of the cluster (1 = urban; 2 = rural).
Region	Region of residence within the country (1 = North Eastern; 2 = North Western; 3 = Central Eastern; 4 = Central; 5 = Central Western; 6 = Southern Eastern; 7 = Southern Western).

### Statistical analyses

Stata 14.0 (Stata Corporation, College Stations, TX, USA) was used to analyze data of this study [15]. Weighted frequency was calculated for all study variables after adjusting for two-stage cluster sampling design of the survey. This analysis included one live-birth per woman as a single unit.

First, a frequency table was used to describe the basic characteristics (Table 2). After reporting the frequency distribution, logistic regression analysis was conducted to investigate the association between the potential determinants and early neonatal deaths. Before conducting the logistic regression analysis, continuous (e.g., maternal age) and discrete variables (e.g., parity) were converted into categorical variables. Crude odds ratios (CORs) were calculated by entering all potential predictors into the baseline equation (i.e., one variable at a time) with early neonatal mortality as the outcome variable (Table 3).

**Table 2:** General characteristics of the study population

Variables	Early neonatal deaths		
	No (N (%))^a^	Yes (N (%))^a^	Total (N (%))^b^
Individual factors			
Sex			
Female	9279 (98.9)	100 (1.1)	9379 (47.8)
Male	10,092 (98.4)	166 (1.6)	10,257 (52.2)
Birth size			
Average	11,629 (99.2)	97 (0.8)	11,726 (61.2)
Smaller than average	2641 (98.5)	39 (1.5)	2680 (14.0)
Larger than average	4660 (97.9)	101 (2.1)	4761 (24.8)
Multiple gestation			
No	19,177 (98.7)	245 (1.3)	19,422 (98.9)
Yes	193 (90.1)	21 (9.9)	214 (1.1)
Maternal factors			
Maternal age			
≤ 18 years	965 (97.9)	20 (2.1)	985 (5.0)
19–34 years	15,004 (98.8)	180 (1.2)	15,184 (77.3)
35–49 years	3401 (98.1)	65 (1.9)	3466 (17.7)
Birth rank			
Primi	2947 (98.8)	35 (1.2)	2983 (15.2)
Second to fourth pregnancy	8766 (99.0)	85 (1.0)	8852 (45.1)
Fifth or higher pregnancy	7656 (98.1)	145 (1.9)	7801 (39.7)
Birth interval			
< 2 years	4778 (98.2)	89 (1.8)	4867 (24.8)
≥ 2 years	11,602 (98.8)	140 (1.2)	11,743 (59.9)
Antenatal factors			
Desire for pregnancy			
Wanted then	17,188 (98.6)	236 (1.4)	17,423 (88.9)
Wanted later	1172 (99.5)	6 (0.5)	1178 (6.0)
Wanted no more	975 (98.6)	13 (1.4)	989 (5.1)
Antenatal care			
No	7322 (98.2)	136 (1.8)	7458 (38.1)
Yes	12,007 (99.0)	117 (1.0)	12,124 (61.1)
Delivery factors			
Delivery assistance			
Unskilled birth attendant	8919 (98.3)	152 (1.7)	9071 (46.3)
Skilled delivery attendant	10,425 (99.0)	104 (1.0)	10,528 (53.7)
Place of delivery			
Home	17,614 (98.3)	181 (1.7)	10,795 (55.0)
Hospital/health centre	8756 (99.0)	85 (1.0)	8841 (45.0)
Socioeconomic characteristics			
Sex of the household head			
Male	19,132 (98.6)	265 (1.4)	19,397 (98.8)
Female	238 (99.7)	1 (0.3)	239 (1.2)
Maternal education level			
No formal education	16,043 (98.5)	240 (1.5)	16,283 (82.9)
Primary	1589 (99.6)	7 (0.4)	1596 (8.1)
Secondary or higher	1738 (98.9)	19 (1.1)	1757 (9.0)
Paternal education level			
No formal education	11,006 (98.4)	174 (1.6)	11,180 (57.6)
Primary	2830 (98.7)	36 (1.3)	2866 (14.8)
Secondary or higher	5317 (99.0)	52 (1.0)	5369 (27.7)
Wealth status			
Poorest	3859 (98.6)	55 (1.4)	3914 (19.9)
Poorer	3890 (98.2)	73 (1.8)	3963 (20.2)
Middle	3953 (98.3)	67 (1.7)	4020 (20.5)
Richer	4012 (98.9)	42 (1.0)	4055 (20.7)
Richest	3656 (99.2)	29 (0.8)	3685 (18.8)
Community level variables			
Antenatal care utilization			
Low	13,979 (98.5)	209 (1.5)	14,189 (72.3)
High	5391 (99.0)	57 (1.0)	5447 (27.7)
Utilization of facility delivery			
Low	10,751 (98.4)	177 (1.6)	10,927 (55.6)
High	8620 (99.0)	90 (1.0)	8710 (44.4)
Community education level			
Low	16,996 (98.5)	256 (1.5)	17,252 (87.9)
High	2374 (99.6)	10 (0.4)	2385 (12.1)
Community poverty level			
Low	5021 (98.6)	73 (1.4)	5094 (25.9)
High	14,349 (98.7)	193 (1.3)	14,543 (74.1)
Place of residence			
Urban	4528 (99.2)	37 (0.8)	4566 (23.3)
Rural	14,842 (98.5)	229 (1.5)	15,071 (76.8)
Region			
North Eastern	2605 (97.7)	60 (2.3)	2665 (13.6)
North Western	3715 (99.4)	21 (0.6)	3736 (19.0)
Central Eastern	1579 (98.3)	28 (1.7)	1607 (8.2)
Central	3475 (99.0)	34 (1.0)	3509 (17.9)
Central Western	3168 (98.9)	34 (1.1)	3202 (16.3)
Southern Eastern	2046 (97.9)	43 (2.1)	2089 (10.6)
Southern Western	2782 (98.4)	44 (1.6)	2826 (14.4)

^a^ Row percentage, ^b^ Column percentage

**Table 3:** Association of early neonatal mortality with risk factors

Variables	COR (95% CI)	AOR (95% CI)
Individual factors		
Sex		
Female	Ref	Ref
Male	1.5* (1.1–2.1)	1.3 (1.0–1.6)
Birth size		
Average	Ref	Ref
Smaller than average	1.8* (1.1–2.9)	1.8** (1.2–2.6)
Larger than average	2.6*** (1.7–3.9)	2.9*** (2.2–3.8)
Multiple gestation		
No	Ref	Ref
Yes	8.6*** (4.7–15.5)	9.3*** (5.7–15.0)
Maternal factors		
Maternal age		
15–18 years	1.8 (0.9–3.6)	1.8* (1.1–3.2)
19–34 years	Ref	Ref
35–49 years	1.6* (1.1–2.3)	1.7*** (1.3–2.3)
Birth rank		
Primi	1.2 (0.8–2.0)	1.1 (0.7–1.7)
Second to fourth pregnancy	Ref	Ref
Fifth or higher pregnancy	1.9*** (1.3–2.9)	1.4 (0.7–2.8)
Birth Interval		
< 2 years	1.5* (1.0–2.3)	1.7*** (1.3–2.2)
≥ 2 years	Ref	Ref
Antenatal factors		
Desire for pregnancy		
Wanted then	Ref	
Wanted later	0.4* (0.2–0.9)	0.7 (0.4–1.3)
Wanted no more	1.0 (0.5–1.9)	0.9 (0.5–1.7)
Antenatal care		
No	Ref	Ref
Yes	0.5*** (0.4–0.7)	0.7* (0.5–0.9)
Delivery factors		
Delivery assistance		
Unskilled birth attendant	Ref	
Skilled delivery attendant	0.6** (0.4–0.8)	
Place of delivery		
Home	Ref	Ref
Hospital/health centre	0.6** (0.4–0.8)	0.7* (0.5–0.9)
Socioeconomic characteristics		
Sex of the household head		
Male	Ref	Ref
Female	0.2* (0.1–0.8)	1.1 (0.3–1.4)
Maternal education level		
No formal education	Ref	Ref
Primary	0.3* (0.1–0.8)	0.6 (0.3–1.1)
Secondary or higher	0.7 (0.4–1.5)	1.6 (0.9–2.7)
Paternal education level		
No formal education	Ref	Ref
Primary	0.8 (0.5–1.2)	0.9 (0.6–1.3)
Secondary or higher	0.6* (0.4–0.9)	0.7* (0.5–1.0)
Wealth status		
Poorest	Ref	Ref
Poorer	1.3 (0.8–2.1)	1.0 (0.7–1.5)
Middle	1.2 (0.7–1.9)	1.2 (0.8–1.8)
Richer	0.7 (0.4–1.3)	1.1 (0.7–1.7)
Richest	0.6^1^ (0.3–1.1)	1.1 (0.6–1.1)
Community level variables		
Antenatal care utilization		
Low	Ref	Ref
High	0.7^1^ (0.5–1.1)	1.0 (0.7–1.4)
Utilization of facility delivery		
Low	Ref	Ref
High	0.6* (0.4–0.9)	1.0 (0.7–1.3)
Community women’s education		
Low	Ref	Ref
High	0.3* (0.1–0.8)	0.4* (0.2–0.9)
Community poverty		
Low	Ref	
High	0.9 (0.6–1.4)	
Place of residence		
Urban	Ref	Ref
Rural	1.9* (1.1–3.2)	1.1 (0.7–1.6)
Region		
North Eastern	Ref	Ref
North Western	0.3*** (0.2–0.6)	0.5** (0.3–0.8)
Central Eastern	0.8 (0.5–1.3)	1.1 (0.7–1.8)
Central	0.5* (0.3–0.9)	0.9 (0.6–1.5)
Central Western	0.6* (0.4–0.9)	0.5* (0.3–0.9)
Southern Eastern	1.0 (0.6–1.7)	1.0 (0.6–1.6)
Southern Western	0.9 (0.5–1.6)	0.4*** (0.2–0.6)

1 - *p* < 0.2, * *p* < 0.05, ** *p* < 0.01, *** *p* < 0.001, *COR* crude odds ratio, *AOR* adjusted odds ratio, *CI* confidence interval

The hierarchical nature of the AfDHS data was considered for the multivariable analysis. Thus, a two-level multivariable analysis was applied. Proximate and socioeconomic factors were considered to be nested within the community (i.e., cluster) level. Covariates with a predetermined significance level (*p* < 0.20) in bivariate analyses were included in the adjustment for multivariable analysis (Table 3) and adjusted odds ratios (AORs) were calculated. To prevent residual confounding in multivariate analysis, the significance level of 0.20 is considered sufficient [16]. Odds ratios (ORs) were reported with 95% confidence intervals (CIs) and significance levels (Table 3). Variance inflation factors were assessed to examine collinearity between variables before entering them into the multivariable models. The AfDHS employed the principal component analysis of basic housing construction materials (i.e., materials used to construct the walls, roofs, and floors), sources of water, sanitation facilities, electricity, and household belongings to construct wealth index score for the households' wealth status. Then the wealth status was stratified into quintiles: poorer, poorest, middle, richer and richest [17, 18].

## Results

### Characteristics of the study sample

Table 2 presents distribution of selected risk factors for early neonatal mortality. Numbers and percentages of the analysis were weighted by the individual sampling of the sampling weight from the AfDHS data. Among the included 19,636 weighted live-births that took place within four years preceding the survey, 1.4% (*n* = 266) children died within the first week of their birth. Approximately 52.2% of the study sample was males. Percentage of neonatal mortality was higher among males (1.6%) compared to females (1.1%). Nearly 61.1% (*n* = 11,727) of the infants were average size at birth and had lower mortality rate than smaller or larger infants. Most of the newborns were singletons, 98.9%. About one-sixth of the mothers were primi (i.e., became pregnant for the first-time in life). A vast majority of the mothers were in the middle age group (19–34 years, 77.3%), followed by older (35–49 years, 17.7%) and younger age groups (≤18 years, 5.0%). A majority of the mothers received antenatal care, 61.9%. Nearly half of the deliveries were conducted by skilled attendants that had a lower percentage of early neonatal mortality in comparison to the deliveries conducted by unskilled attendants, 1.0% and 1.7%, respectively. About 55.0% of the deliveries were conducted at home. Most of the households’ head were males. A vast majority of the mothers (82.9%) had no formal education. Approximately an equal number of respondents were obtained from each household wealth quintile group or each region of the country. More than three-fourths of the participants were from rural areas. About one-fourth of the participants were from a community where at least 25% of the women received four or more antenatal visits. Most of the infants were from a community with ‘low’ education level (87.9%), which in turn had a higher percentage of neonatal deaths than the communities with higher education level, 0.4% and 1.4%, respectively.

### Determinants of early neonatal mortality

Table 3 shows results of logistic regression analyses. In unadjusted level, multiple gestations had the highest odds of early neonatal mortality (COR: 8.6; 95% CI: 4.7–15.5). Male children had a higher likelihood of dying than their female counterparts (COR: 1.6; 95% CI: 1.1–2.1). Both smaller and larger babies had higher odds of mortality than average sized babies. Birth rank was found to be associated with a higher likelihood of mortality among a birth order of five or more (COR: 1.9; 95% CI: 1.3–2.9). Children delivered by mothers with a birth interval of less than two years had higher odds of dying than a birth interval of two or more years (COR: 1.5; 95% CI: 1.0–2.3). Maternal age also had a significant association with the outcome variable. Children delivered by mothers who received antenatal care (COR: 0.5; 95% CI: 0.4–0.7), delivered by skilled health personnel (COR: 0.6; 95% CI: 0.4–0.8) and delivered at heath facilities (COR: 0.6; 95% CI: 0.4–0.8) were less likely to die than the children delivered by mothers who did not utilize these services. Parental education levels also had a significant association with the outcome variable. Rural infants were nearly two times more likely to die than urban infants (COR: 1.9; 95% CI: 1.1–3.2). Odds of deaths were significantly lower in north-western (COR: 0.3; 95% CI: 0.2–0.6) and central-western (COR: 0.6; 95% CI: 0.4–0.9) regions than the north-eastern part.

We found two pairs of collinear variables: parity and delivery attendance with maternal age and place of delivery, respectively; we kept maternal age and place of delivery for adjustment. After adjusting the variables, ordered from the most significant odds, the following categories were significantly associated with an increased likelihood of early neonatal mortality: multiple gestations (AOR: 9.3; 95% CI: 5.7–15.0), larger (AOR: 2.9; 95% CI: 2.2–3.8) than average birth size, birth interval of < 2 years (AOR: 2.6; 95% CI: 1.4–4.9), smaller (AOR: 1.8; 95% CI: 1.2–2.6) than average birth size, maternal age ≤ 18 years (AOR: 1.8; 95% CI: 1.1–3.2) and ≥ 35 years (AOR: 1.7; 95% CI: 1.3–2.3). The variables associated with lower odds of dying were: antenatal care by a skilled provider (AOR: 0.7; 95% CI: 0.5–0.9), institutional delivery (AOR: 0.7; 955 CI: 0.5–0.9), paternal higher education level (AOR: 0.7; 95% CI: 0.5–1.0), living in north-western (AOR: 0.3; 95% CI: 0.1–0.6), central-western regions (AOR: 0.5; 95% CI: 0.3–0.9) or in a community with higher maternal education level (AOR: 0.4; 95% CI: 0.2–0.9).

## Discussion

We examined the determinants of early neonatal mortality in Afghanistan and found that birth size, multiple gestations, shorter birth interval, and younger (≤18 years) or older (≥35 years) maternal age were associated with higher likelihood of early neonatal mortality. On the other hand, antenatal care during pregnancy, facility delivery, paternal education level, and higher education level of women in the community had a protective effect against early neonatal mortality. We have reconfirmed the significance of these known risk factors for early neonatal deaths in the context of this country. To the best of our knowledge, this is the first epidemiological study which examined determinants of early neonatal mortality in Afghanistan.

Multiple gestations are a known risk factor for neonatal deaths in developing countries; earlier reports from several lower and middle-income countries found higher risks of deaths among infants of multiple gestations [19–21]. The explanation that has been put forward for this higher risk of dying is that multiple births have a higher proportion of prematurity which is one of the major causes of neonatal deaths. Prematurity predisposes infants to a higher risk of infection, hypoglycemia, and hypothermia [22]. These co-morbid conditions could cause a baby to become critically ill which could be too difficult of a condition to manage in low resource settings in a developing country like Afghanistan [4].

Similar to earlier studies, birth size was a significant predictor [11, 23, 24]; both smaller and larger birth size had positive associations with early neonatal mortality. Smaller than average birth size children could be a proxy for the low birth weight babies and may result from premature births. As explained earlier, this could predispose these infants to infections and other abnormalities [22]. On the other hand, larger babies have a higher risk of birth injury, respiratory distress due to birth asphyxia and congenital anomaly which could contribute to the higher likelihood of early neonatal deaths [25, 26].

Children with shorter (< 2 years) birth intervals were inversely associated with early neonatal survival. This finding is consistent with previous studies [23, 24, 27]. A longitudinal study from Bangladesh found higher risks of deaths among newborns with a birth interval of two or less years compared to a birth interval of three or more years [28]. This finding indicates that prolongation of the interval between two subsequent pregnancies helps to prepare the mother for the later pregnancy. Evidence also suggests that adequate supply of essential nutrients is ensured during this prolonged period of birth interval [29].

Women who received antenatal care during pregnancy were less likely to experience death of their offspring than the women who did not utilize this service; this is also confirmed by earlier reports [30, 31]. Antenatal care has been recommended as one of the four main pillars of the ‘Safe Motherhood Initiative’ based on its effectiveness [32]. It improves pregnancy outcomes by identifying and managing most pregnancy complications. With the identification and management of pregnancy complications, pregnant women receive counseling about the importance of safe delivery practices and early management of newborns’ illness [33, 34]. This study also found that infants born of women who lived in a community where at least one-fourth of the women received antenatal visits had better survival in the early neonatal period. Currently, the World Health Organization (WHO) recommends eight or more antenatal care visits for uncomplicated pregnancies [35]; ensuring these visits is crucial for better survival of neonates. However, this study found that more than one-third of the women did not receive a single antenatal care visit during pregnancy, indicating that this important service is severely under-utilized in this country.

Our results also found positive associations between facility delivery and neonatal survival, which is similar to findings in previous studies from other countries [30, 36], however several previous studies found no association between institutional delivery and early neonatal survival [12, 37–39] or higher likelihood of deaths among hospital-born babies [40]. This higher likelihood or insignificant relationship could be due to increased association between delivery complications and institutional deliveries [41–43]. In addition to management of delivery complications, newborns could be benefitted from primary interventions in a health facility [44]. The ‘Three Delays Model’ implies that delays in recognizing and reaching care could cause adverse outcomes due to delays in receiving care [45, 46]. Though that model was developed for care seeking of maternal illness, it could be appropriate in this context as well in a way that facility delivery could minimize the time required for the first ‘two’ delays which are crucial for immediate management and survival of newborns [45, 46].

Maternal age also significantly influenced the odds of dying; this finding is ubiquitous across settings [23, 24, 31, 47]. Association of younger age could be due to the fact that the mother did not reach her full physical or reproductive maturity for child bearing. In addition to this physical or reproductive immaturity, lack of experience related to child-care could also be a contributing factor [48]. Infants delivered by younger mothers are prone to being born premature, and having low birth weight and congenital malformations [49]. The association between early neonatal mortality and late maternal age could be due to higher risk of delivering high or low birth weight babies among older mothers [50]. In addition to this, advanced maternal age is associated with antenatal and delivery complications [51]; these complications are known risk factors for early neonatal deaths [20, 52].

Although the gross difference of rural-urban residence was eliminated after adjusting for other included factors, this study found regional variation in early neonatal mortality; this is consistent with reports from other countries [23, 24, 30, 37, 53]. The AfDHS also concluded that there are substantial variations in maternal health services utilization in different parts of this country; these differences could contribute to the overall differences in early neonatal mortality among regions of this country. However, this finding points to the need for prioritizing the regions with high newborn mortality rates.

Several non-significant factors also warrant further discussions. In addition to the place of residence, this study did not find any association of maternal education, and household wealth status with early neonatal mortality. Though previous studies from different countries found associations of socioeconomic conditions with early neonatal deaths [20, 37], recent analyses from several other studies found that socioeconomic inequalities in neonatal mortality in low and middle-income countries are falling. The explanation that has been put forward for the reduction of these inequalities is that the ‘diminution of inequalities’ associated with maternal and neonatal health service utilization [38, 54, 55].

This study has several notable strengths. The foremost strength of this study is that it is generalizable for the entire country of Afghanistan, as it covered both the urban and rural areas in all thirty-four provinces of the country. The sample size of this population-based survey was also large (*n* = 19,801). The response rate was approximately 97% and missing data was very low. The inclusion of only the last live-birth within four years preceding the survey reduced recall bias. The conceptual framework that we used has also been used by earlier studies that investigated determinants of child-hood mortality in similar settings  [14, 23, 24]. Additionally, trained field-staff, standardized measurement methodologies and tools increased authenticity of our findings [4].

Despite the above-mentioned strengths, limitations of the current study also merit discussions. Due to limitations of the dataset, we were unable to investigate some determinants such as maternal nutrition, environmental, and genetic factors which are also associated with childhood survival [14]. We only analyzed data of survived women, therefore excluding determinants of the more adversely affected mothers may lead to an underestimation of the burden early neonatal mortality. The deaths were reported only based on maternal reports, not confirmed by verbal autopsy – which could be subject to recall bias in addition to misclassification of some stillbirths as early neonatal deaths [4]. This cross-sectional survey dataset contains socioeconomic conditions at the time of the survey and included live-births within four years of the survey; causality cannot be established due to uncertainty about temporal association. Maternal report about size at birth may result in some misclassification due to recall bias or subjective assessment [4].

### Recommendations

An integrated or multifaceted approach is required to address all potential factors of various levels associated with early neonatal deaths in Afghanistan. From a program-planning perspective, it is essential to take modifiable risk factors into account to design newborns’ survival program. Implementation of the continuum of care approach is required to further increase the proportion of women with antenatal care, facility delivery or delivery by skilled delivery personnel [56]. Adapting family planning services for women to increase birth interval timing could reduce the proportion of pregnancy among younger or older mother. Delaying age at marriage could also reduce mortality, which is associated with younger maternal age [49]. To reduce premature births, several behavioral (e.g., smoking cessation) and medical interventions (e.g., progesterone supplementation) are effective [57]. Moreover, Kangaroo Mother Care (KMC) could be an effective and low-cost intervention to prevent preterm or small size newborns’ mortality [58, 59]. These components should be included in newborns’ survival strategy. Furthermore, strengthening of the Afghan health system could facilitate better management of preterm or larger babies in addition to other sick newborns, as well as increase utilization and quality of such services. Further research is required to recognize other unexamined factors in the context of this country. A common platform could be made with researchers and policymakers from all South Asian countries, as most of the countries in this region share this common problem of high early neonatal mortality rates [1, 2].

## Conclusions

This study investigated a nationally representative dataset from Afghanistan to identify determinants of early neonatal mortality and reconfirmed association of individual, maternal, antenatal, and socioeconomic factors with survival in this age group. In addition to identification of these determinants, this study recommended taking a comprehensive approach to addressing these associated factors of multiple levels; any neonatal survival program needs to incorporate antenatal, delivery and family planning services to prevent early neonatal mortality in addition to proper management of preterm and sick newborns.

## Abbreviations

AfDHS: Afghanistan Demographic and Health Survey
AOR: Adjusted odds ratio
ARR: Annual rate of reduction
CI: Confidence interval
COR: Crude odds ratio
EA: Enumeration area
NMR: Neonatal mortality rate
OR: Odds ratio
SDG: Sustainable Development Goals
UN: United Nations
VIF: Variance inflation factor
WHO: World Health Organization

## References

[1] You D, Hug L, Ejdemyr S, Idele P, Hogan D, Mathers C, Gerland P, New JR, Alkema L, United Nations Inter-agency Group for Child Mortality Estimation (UN IGME) (2015). Global, regional, and national levels and trends in under-5 mortality between 1990 and 2015, with scenario-based projections to 2030: a systematic analysis by the UN inter-agency Group for Child Mortality Estimation. Lancet.

[2] UN Inter–agency Group for Child Mortality (2015). Child Mortality Estimates September 2015.

[3] United Nations, Sustainable Development Goals. http://www.un.org/sustainabledevelopment/health/. Accessed 6 May 2017.

[4] Central SO, Ministry of PH, ICF (2017). Afghanistan Demographic and Health Survey 2015.

[5] Central Intelligence Agency - The World Factbook. https://www.cia.gov/library/publications/the-world-factbook/geos/af.html. Accessed 6 May 2017.

[6] Akseer N, Lawn JE, Keenan W, Konstantopoulos A, Cooper P, Ismail Z, Thacker N, Cabral S, Bhutta ZA (2015). Ending preventable newborn deaths in a generation. Int J Gynaecol Obstet.

[7] Bhutta ZA, Das JK, Bahl R, Lawn JE, Salam RA, Paul VK, Sankar MJ, Blencowe H, Rizvi A, Chou VB, Walker N, Lancet Newborn Interventions Review Group, Lancet Every Newborn Study Group (2014). Can available interventions end preventable deaths in mothers, newborn babies, and stillbirths, and at what cost?. Lancet.

[8] Sankar MJ, Natarajan CK, Das RR, Agarwal R, Chandrasekaran A, Paul VK (2016). When do newborns die? A systematic review of timing of overall and cause-specific neonatal deaths in developing countries. J Perinatol.

[9] Oza S, Cousens SN, Lawn JE (2014). Estimation of daily risk of neonatal death, including the day of birth, in 186 countries in 2013: a vital-registration and modelling-based study. Lancet Glob Health.

[10] Fawole AO, Shah A, Tongo O, Dara K, El-Ladan AM, Umezulike AC, Alu FE, Eniayewun AB, Fabanwo AO, Adewunmi AA, Adegbola O, Adebayo AA, Obaitan FO, Onala OE, Usman Y, Sullayman AO, Kailani S, Sa’id M (2011). Determinants of perinatal mortality in Nigeria. Int J Gynaecol Obstet.

[11] Smeeton NC, Rona RJ, Dobson P, Cochrane R, Wolfe C (2004). Assessing the determinants of stillbirths and early neonatal deaths using routinely collected data in an inner city area. BMC Med.

[12] Nankabirwa V, Tumwine JK, Tylleskar T, Nankunda J, Sommerfelt H, PROMISE EBF Research Consortium (2011). Perinatal mortality in eastern Uganda: a community based prospective cohort study. PLoS One.

[13] Tachiweyika E, Gombe N, Shambira G, Chadambuka A, Mufuta T, Zizhou S (2011). Determinants of perinatal mortality in Marondera district, Mashonaland East Province of Zimbabwe, 2009: a case control study. Pan Afr Med J.

[14] Mosley WH, Chen LC (2003). An analytical framework for the study of child survival in developing countries. 1984. Bull World Health Organ.

[15] Stata Corporation (2015). Stata Statistical Software. Release 14.0.

[16] Maldonado G, Greenland S (1993). Simulation study of confounder-selection strategies. Am J Epidemiol.

[17] Jolliffe IT, Cadima J (2016). Principal component analysis: a review and recent developments. Philos Trans A Math Phys Eng Sci.

[18] Filmer D, Pritchett LH (2001). Estimating wealth effects without expenditure data--or tears: an application to educational enrollments in states of India. Demography.

[19] Jahn A, Kynast-Wolf G, Kouyate B, Becher H (2006). Multiple pregnancy in rural Burkina Faso: frequency, survival, and use of health services. Acta Obstet Gynecol Scand.

[20] Owais A, Faruque AS, Das SK, Ahmed S, Rahman S, Stein AD (2013). Maternal and antenatal risk factors for stillbirths and neonatal mortality in rural Bangladesh: a case-control study. PLoS One.

[21] Kayode GA, Ansah E, Agyepong IA, Amoakoh-Coleman M, Grobbee DE, Klipstein-Grobusch K (2014). Individual and community determinants of neonatal mortality in Ghana: a multilevel analysis. BMC Pregnancy Childbirth.

[22] Lawn J, Kerber K (2006). Opportunities for Africas newborns: practical data policy and programmatic support for newborn care in Africa.

[23] Nisar YB, Dibley MJ (2014). Determinants of neonatal mortality in Pakistan: secondary analysis of Pakistan Demographic and Health Survey 2006–07. BMC Public Health.

[24] Titaley CR, Dibley MJ, Agho K, Roberts CL, Hall J (2008). Determinants of neonatal mortality in Indonesia. BMC Public Health.

[25] Ng SK, Olog A, Spinks AB, Cameron CM, Searle J, McClure RJ (2010). Risk factors and obstetric complications of large for gestational age births with adjustments for community effects: results from a new cohort study. BMC Public Health.

[26] Sjaarda LA, Albert PS, Mumford SL, Hinkle SN, Mendola P, Laughon SK (2014). Customized large-for-gestational-age birthweight at term and the association with adverse perinatal outcomes. Am J Obstet Gynecol.

[27] Rutstein SO (2005). Effects of preceding birth intervals on neonatal, infant and under-five years mortality and nutritional status in developing countries: evidence from the demographic and health surveys. Int J Gynaecol Obstet.

[28] DaVanzo J, Hale L, Razzaque A, Rahman M (2008). The effects of pregnancy spacing on infant and child mortality in Matlab, Bangladesh: how they vary by the type of pregnancy outcome that began the interval. Popul Stud (Camb).

[29] Zenger E (1993). Siblings’ neonatal mortality risks and birth spacing in Bangladesh. Demography.

[30] Rahman MM, Abidin S (2010). Factors affecting neonatal mortality in Bangladesh. J Health Manag.

[31] Kananura RM, Tetui M, Mutebi A, Bua JN, Waiswa P, Kiwanuka SN, Ekirapa-Kiracho E, Makumbi F (2016). The neonatal mortality and its determinants in rural communities of Eastern Uganda. Reprod Health.

[32] World Health Organization (1996). Mother-Baby Package: Implementing safe motherhood in countries. Practical Guide.

[33] Bloom SS, Lippeveld T, Wypij D (1999). Does antenatal care make a difference to safe delivery? A study in urban Uttar Pradesh, India. Health Policy Plan.

[34] Institute of Medicine (US) Committee on Improving Birth Outcomes. Improving Birth Outcomes: Meeting the Challenge in the Developing World. Washington: National Academies Press (US); 2003. http://www.ncbi.nlm.nih.gov/books/NBK222097/. Accessed 26 Apr 2017. 25057689

[35] Tuncalp P-RJP, Lawrie T, Bucagu M, Oladapo OT, Portela A, Metin Gulmezoglu A (2017). WHO recommendations on antenatal care for a positive pregnancy experience-going beyond survival. BJOG.

[36] Yirgu R, Molla M, Sibley L, Gebremariam A (2016). Perinatal mortality magnitude, determinants and causes in west Gojam: population-based nested case-control study. PLoS One.

[37] Kumar C, Singh PK, Rai RK, Singh L (2013). Early neonatal mortality in India, 1990-2006. J Community Health.

[38] Engmann C, Walega P, Aborigo RA, Adongo P, Moyer CA, Lavasani L, Williams J, Bose C, Binka F, Hodgson A (2012). Stillbirths and early neonatal mortality in rural northern Ghana. Tropical Med Int Health.

[39] Bari W, Chowdhury RI, Islam MA, Chakraborty N, Akhter HA (2002). The differentials and determinants of perinatal mortality in rural Bangladesh. Eur J Contracept Reprod Health Care.

[40] Fikree FF, Gray RH (1996). Demographic survey of the level and determinants of perinatal mortality in Karachi, Pakistan. Paediatr Perinat Epidemiol.

[41] Sarker BK, Rahman M, Rahman T, Hossain J, Reichenbach L, Mitra DK (2016). Reasons for preference of home delivery with traditional birth attendants (TBAs) in rural Bangladesh: a qualitative exploration. PLoS One.

[42] Belda SS, Gebremariam MB (2016). Birth preparedness, complication readiness and other determinants of place of delivery among mothers in Goba District, Bale Zone, South East Ethiopia. BMC Pregnancy Childbirth.

[43] Liambila WN, Kuria SN (2014). Birth attendance and magnitude of obstetric complications in Western Kenya: a retrospective case-control study. BMC Pregnancy Childbirth.

[44] Moyer CA, Dako-Gyeke P, Adanu RM (2013). Facility-based delivery and maternal and early neonatal mortality in sub-Saharan Africa: a regional review of the literature. Afr J Reprod Health.

[45] Waiswa P, Kallander K, Peterson S, Tomson G, Pariyo GW (2010). Using the three delays model to understand why newborn babies die in eastern Uganda. Tropical Med Int Health.

[46] Barnes-Josiah D, Myntti C, Augustin A (1998). The “three delays” as a framework for examining maternal mortality in Haiti. Soc Sci Med.

[47] Lisonkova S, Janssen PA, Sheps SB, Lee SK, Dahlgren L (2010). The effect of maternal age on adverse birth outcomes: does parity matter?. J Obstet Gynaecol Can.

[48] Bicego G, Ahmad O (1992). Infant and child mortality. Pakistan Demographic and Health Survey.

[49] Kang G, Lim JY, Kale AS, Lee LY (2015). Adverse effects of young maternal age on neonatal outcomes. Singap Med J.

[50] Kenny LC, Lavender T, McNamee R, O'Neill SM, Mills T, Khashan AS (2013). Advanced maternal age and adverse pregnancy outcome: evidence from a large contemporary cohort. PLoS One.

[51] Berkowitz GS, Skovron ML, Lapinski RH, Berkowitz RL (1990). Delayed childbearing and the outcome of pregnancy. N Engl J Med.

[52] Khanam R, Ahmed S, Creanga AA, Begum N, Koffi AK, Mahmud A, Rosen H, Baqui AH, Projahnmo Study Group in Bangladesh (2017). Antepartum complications and perinatal mortality in rural Bangladesh. BMC Pregnancy Childbirth.

[53] Mekonnen Y, Tensou B, Telake DS, Degefie T, Bekele A (2013). Neonatal mortality in Ethiopia: trends and determinants. BMC Public Health.

[54] McKinnon B, Harper S, Kaufman JS, Bergevin Y (2014). Socioeconomic inequality in neonatal mortality in countries of low and middle income: a multicountry analysis. Lancet Glob Health.

[55] Berhan Y, Berhan A (2014). A meta-analysis of socio-demographic factors for perinatal mortality in developing countries: a subgroup analysis of the national surveys and small scale studies. Ethiop J Health Sci.

[56] Kerber KJ, de Graft-Johnson JE, Bhutta ZA, Okong P, Starrs A, Lawn JE (2007). Continuum of care for maternal, newborn, and child health: from slogan to service delivery. Lancet.

[57] Chang HH, Larson J, Blencowe H, Spong CY, Howson CP, Cairns-Smith S, Lackritz EM, Lee SK, Mason E, Serazin AC, Walani S, Simpson JL, Lawn JE, Born Too Soon preterm prevention analysis group (2013). Preventing preterm births: analysis of trends and potential reductions with interventions in 39 countries with very high human development index. Lancet.

[58] Suman RP, Udani R, Nanavati R (2008). Kangaroo mother care for low birth weight infants: a randomized controlled trial. Indian Pediatr.

[59] Conde-Agudelo A, Diaz-Rossello JL (2014). Kangaroo mother care to reduce morbidity and mortality in low birthweight infants. Cochrane Database Syst Rev.

